# Alternate Sequential Suture Tightening: A Novel Technique for Uncontrolled Postpartum Hemorrhage

**DOI:** 10.1155/2015/145178

**Published:** 2015-03-22

**Authors:** Sharda Brata Ghosh, Y. M. Mala

**Affiliations:** ^1^Saudi German Hospital, Dubai, UAE; ^2^Department of Obstetrics & Gynaecology, Lok Nayak Hospital, Maulana Azad Medical College, Delhi 110002, India

## Abstract

*Objective*. The most commonly described technique of modified B-Lynch suture may not be suitable for all the patients presenting with flabby, atonic uterus.* Study Design*. A retrospective analysis of twelve patients with uncontrolled postpartum haemorrhage, who underwent this procedure from March 2007 to September 2012, was conducted. In this novel technique, sutures are passed in the lower uterine segment and are tightened alternately to control uterine bleeding.* Results*. Average duration of the procedure was 4 minutes (range 2–7 minutes). Average blood loss was 1625 mL (range 1300–1900 mL). Eleven patients (91.66%) were seen to have a successful outcome with only this technique. No patient required hysterectomy and one patient (8.33%) required additional bilateral internal iliac artery ligation. All the patients had a minimum follow-up of 2 yrs and none of them reported any infertility problems.* Conclusion*. This technique is simple, quick, and effective. There was no adverse effect on the fertility potential for the observed 2 years; however, a long-term follow-up is required to comment on its actual rate. This technique cannot replace the standard modified B-Lynch technique for uncontrolled postpartum haemorrhage but can be used for unresponsive, flabby, and atonic uterus.

## 1. Introduction

Postpartum haemorrhage remains one of the leading causes of maternal morbidity and mortality [1]. In developed countries, it accounts for 0.1% of maternal deaths [2] and is responsible for approximately yearly deaths of around 125,000 women all over the world [3]. For more than a decade, modified B-Lynch suture has been the standard technique for managing uncontrolled postpartum haemorrhage [4–6], but, in some patients, it does not have a successful outcome. In developing countries, this may lead to emergency hysterectomy or maternal mortality due to inadequate medical facilities. In order to avoid or reduce the rate of such avoidable complications, we have tried to modify the original technique of modified B-Lynch suture and present our results as seen in the operated patients of uncontrolled PPH.

## 2. Material and Methods

A retrospective study was conducted on all the women who were diagnosed with postpartum haemorrhage from March 2007 up to September 2012. The data was analyzed regarding total number and mode of deliveries, number of cases of postpartum haemorrhage, their complications, and duration of hospital stay. Blood loss was estimated based on the swab count (one small 10 × 10 cm fully saturated swab: around 60 mL of blood loss; one large 45 × 45 cm fully saturated swab: around 350 mL blood loss). Patients having PPH after vaginal delivery and PPH without uterine atony were excluded from the study.

This technique was described and developed by the corresponding author. It was used as a secondary procedure in the first patient following a failed modified B-Lynch suture and then as a primary procedure in the other eleven patients (Table 1). Primary indication in these 11 patients was persistent atonic uterus which did not respond to other conservative measures including uterotonics (I/V oxytocin, ergometrine, carboprost, and misoprostol) and uterine massage. Uterotonic treatment included bolus dose of 5 units of the oxytocin followed by infusion of 20 units of oxytocin; 0.2 mg of ergometrine was given and repeated 2-3 times; 250 micrograms of carboprost was given by intramyometrial route and was repeated 2-3 times; 1000 micrograms of the misoprostol was given rectally. All the patients were discharged after a minimum of 8 days of hospital stay and were followed up regularly.

## 3. Alternate Sequential Suture Tightening (ASST) Technique 

### 3.1. Position and Technique

After induction of anaesthesia, patient is positioned, cleaned, and draped for lower segment caesarean section. After delivery of the baby, if atonic PPH is observed, medical management (as described in Section 2) and uterine massage are started. If patient does not respond to this treatment, then resuscitation is started along with the arrangement and transfusion of adequate blood and blood products. If medical treatment fails then the uterus is exteriorized and bimanual compression is applied to assess the feasibility of the Alternate Sequential Suture Tightening (modified B-Lynch) technique. Two number 2 Vicryl sutures preferably on straight needles are mounted. First needle (right side) is inserted into the uterus above (1-2 cm) the bladder reflection and 1-2 cm medial to the lateral edge of lower uterine segment and 1–3 cm below the lower uterine incision. Then needle from this point of insertion into the anterior wall is taken out from the posterior wall at the same level. Then both free ends of the sutures are tied by a knot (double throw) at the fundus of the uterus. Then the knot is held by artery forceps. Similarly, another suture is applied and tied over the fundus and is held with artery forceps on the left side. Then artery forceps on the right side are opened and the suture is tightened further and again forceps are held at further tightened point. Now similar procedure is repeated on the left side. Such alternative sequential tightening of both sutures is repeated 3-4 times till the uterus is completely compressed and bleeding is controlled. Both sutures can be tightened up to around 3–5 cm of their extra length from the initial level of tightening by this Alternate Sequential Suture Tightening technique. Finally, one square knot is applied on both sides to lock the sutures. Now incision line is observed for any bleeding; if there is no bleeding, uterus is closed in the regular manner.

### 3.2. Postoperative Management

All the patients were monitored for hypovolemia and contracted uterus. Vulval examination was done to detect any vaginal bleeding. All the patients were discharged after a minimum of 8 days of hospital stay. Patients were followed up regularly at 1.5, 3, 6, 12, and 24 months after the index procedure.

## 4. Results

A total of 199 patients had atonic postpartum haemorrhage of which 174 patients developed it after vaginal deliveries and 25 patients after caesarean sections. Out of the 25, thirteen patients responded to uterotonics with uterine massage; the remaining twelve patients did not respond and hence were managed by this technique. Average duration of the procedure was 4 minutes (range 2–7 minutes). Average blood loss was 1625 mL (range 1300–1900 mL). Average duration of hospital stay was 10 days (range 8–16 days).

Eleven patients (91.66%) were seen to have a successful outcome with this technique alone. No patient required hysterectomy and one patient (8.33%) required additional bilateral internal iliac artery ligation. Two patients had postoperative fever for 3 days while two had mild superficial infection, all of whom recovered well with conservative treatment. All the patients had minimum follow-up of 2 yrs and none of them reported any infertility problems.

## 5. Discussion

Postpartum haemorrhage is a potentially life threatening complication associated with foetal delivery [1]. It may occur after vaginal delivery (4%) or caesarean section (6%) [7, 8]. It is usually unpredictable and remains a challenge for obstetricians worldwide. In the developing world, due to factors such as high prevalence of high risk pregnant women, nonavailability of blood products and/or of operation theatre/intensive care unit back-up may further complicate the situation. Risk factors for PPH include anemia, uncontrolled hypertension, multigravida mothers, multiple pregnancies, coagulopathy, previously scared uterus, prolonged labor, and instrumental delivery [2, 7, 8]. Some common underlying causes include uterine atony, retained conception product, abnormal placental implantation, uterogenital trauma, and undetected coagulopathies [9].

The most important step in the management of PPH is to identify the underlying cause and correct the same [4]. Most of the cases of PPH can be controlled by traditional treatment modalities like uterotonic agents, uterine massage, and balloon tamponade [4]. Uncontrolled PPH is usually managed by different uterine suture techniques (B-Lynch, modified B-Lynch, and square suture) or with stepwise devascularization surgical procedures. These techniques have reported variable outcomes and many of the patients finally require emergency hysterectomy [4, 5, 10, 11].

B-Lynch suture was first described and was shown to be the most effective suture technique for managing uncontrolled postpartum haemorrhage due to atonic uterus. But it requires expertise and reopening of the uterus. As a result, a lot of complications (partial necrosis or sloughing of the uterine wall, cervical stenosis, and hematometra) have been reported in literature [12]. Hayman et al. [5] have performed a modified original B-Lynch suture independently. They did not reopen the uterine cavity and used number 2 Vicryl or Dexon suture to control the bleeding. Sutures are taken on long straight needle to transfix the uterus from front to back, just above the reflection of the bladder, and are then tied at the fundus of the uterus. Since then, modified suture has become one of the most popular and standard treatments for managing uncontrolled postpartum haemorrhage [5, 6, 13].

This technique is also used to control massive uterine bleeding due to abnormal placental implantations, midtrimester abortion, and patients with coagulation disorders [5, 12, 13]. A number of suture materials like Dexon (polyglycolic acid), Vicryl (polyglactin 910), PDS (polydioxanone), Prolene (monofilament polypropylene), and nylon have been used for applying these sutures [14, 15]. Many centers use a specially designed Monocryl (poliglecaprone 25, Ethicon) monofilament suture with a 60% tensile strength at 7 days, 0% at 21 days, and complete absorption at 90–120 days [14, 15]. Kaoiean [16] reported successful outcome of B-Lynch suture in 23 patients out of total 24 patients. One patient did not respond and was managed by emergency hysterectomy. Xiao and Zhang reported successful outcome in a patient with PPH managed with combination of B-Lynch and modified Cho suture [17]. Marasinghe et al. [18] did a prospective observational study to evaluate the performance of a modified anchored B-Lynch suture for postpartum atonic uterus in 17 women. They reported successful outcomes in 13 patients (76%) while 4 patients (24%) required emergency postpartum hysterectomy. Kayem et al. [19] did a prospective population based study to assess maternal outcomes after application of uterine compression suture and to characterize the risk factors for obstetric hysterectomy. Two hundred eleven women were managed with a uterine compression suture to control postpartum haemorrhage. The overall rate of failure, leading to hysterectomy, was 25%. However, there were no significant differences in failure rates among B-Lynch sutures, modified B-Lynch sutures, and other suture techniques. They reported that women were more likely to have a hysterectomy if they were aged 35 years or older, were multiparous, had a vaginal delivery, or had a delay between 2 and 6 hours from delivery to uterine suture compression. So, a single or combination of these suture techniques has produced variable results in different published studies. In many patients, uterus may remain flabby which may lead to emergency hysterectomy or maternal mortality due to inadequate medical facilities. Such difficult situations forced us to incorporate some changes in the modified B-Lynch technique.

**Figure 1:**
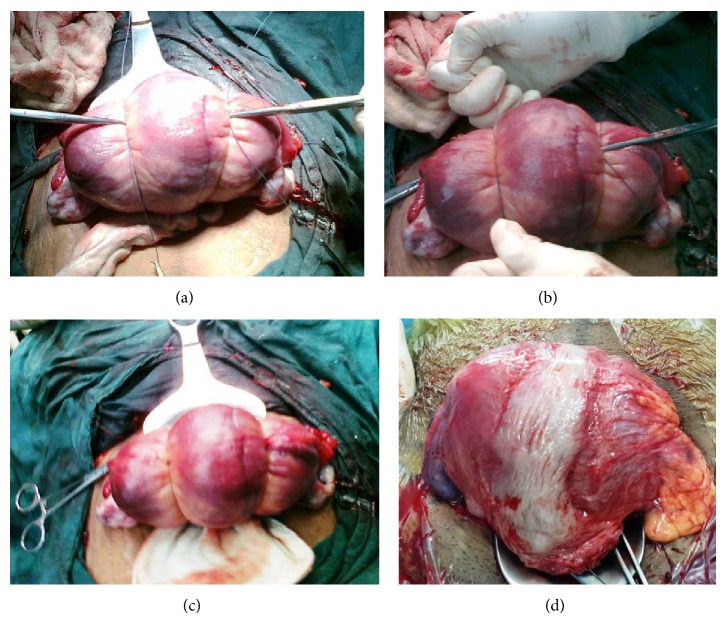
(a) Clinical photograph showing the first level of double tie knot being held with two artery forceps. (b) Clinical photograph showing artery forceps holding the tightened knot on Lt side and the Rt side which is being further tightened (with reduction in the size of uterus). (c) Clinical photograph showing both knots after final tightening of both sutures with marked reduction in the size of uterus. (d) Clinical photograph showing the uterus at 1.5 yrs follow-up during second cesarean section.

This modified technique (Alternate Sequential Suture Tightening) was used as a secondary procedure in the first patient for failed modified B-Lynch suture and then as a primary procedure in the other eleven patients. We believe that uterus remained flabby in some cases, because it could not be compressed fully by the standard modified B-Lynch suture technique. But when we used this technique, uterus could be compressed more effectively and bleeding could be controlled. We used number 2 Vicryl sutures in all of our patients due to unavailability of the Monocryl (poliglecaprone 25, Ethicon) suture. Two number 2 Vicryl sutures, preferably on straight needles, were used in this technique. The first needle (right side) is inserted into the uterus above (1-2 cm) bladder reflection, 1-2 cm medial to the lateral edge of lower uterine segment, and 1–3 cm below the lower uterine incision. Then needle from this point of insertion into the anterior wall is taken out from the posterior wall at the same level. Then both free ends of the sutures are tied by a knot (double throw) at the fundus of the uterus. Then the knot is held by artery forceps (Figure 1). Similarly, another suture is applied and tied over the fundus and is held with artery forceps on the left side. Then artery forceps on the right side are opened and the suture is tightened further and again forceps are held at further tightened point. Now similar procedure is repeated on the left side. Such alternative sequential tightening of both sutures is repeated 3-4 times till the uterus is completely compressed and bleeding is controlled. Finally, one square knot is applied on both sides to lock the sutures.

Almost 75–100% successful outcomes have been reported with modified B-Lynch suture in published literature [18, 19]. But, in our study, eleven patients (91.66%) had successful outcome with our technique only. No patient required hysterectomy and only one patient (8.33%) required additional bilateral internal artery ligation. All the patients had minimum follow-up of 2 yrs and none of them reported any infertility problems. Weakness of this study includes retrospective nature, lack of control, and short follow-up.

This technique is simple, quick, and effective (91.66% successful outcome). There was no adverse effect on the fertility potential for the observed 2 years; however, a long term follow-up is required to comment on its actual rate. This technique cannot replace the standard B-Lynch [20] or modified B-Lynch technique for uncontrolled postpartum haemorrhage but can be used for unresponsive, flabby, and atonic uterus.

## Figures and Tables

**Table 1:** Showing diagnoses, treatment, complications, blood loss, and hospital stay.

Age of patient (yrs)	Diagnosis	Preop Hb (gm%)	Technique	O	Estimated blood loss (mL)	Postop Hb (gm%)	Number of units of blood transfusion	Hospital stay (days)	Complications
29	G2P1L1 37 wks with fetal distress	8.4	**Failed MBLS;** ASTS	S	1400	8.9	2	10	Fever for 3 days

24.5	Primi. with 40 weeks with thick meconium in early labor	7.8	Failed MT ASTS	S	1900	8.3	3	8	—

35	G5P4L4 with 36 wks with APH (PP IIb)	9.7	Failed MT ASTS	S	1600	9.2	1	16	—

33	G3A2 with 41 weeks with PROM	9.4	Failed MT ASTS	S	1800	9.3	2	10	—

29	G3A2 with 41 weeks with CPD	8.9	Failed MT ASTS	S	1500	9.6	2	8	Superficial infection

31	G3A2 with 37 wks with gestational HTN with failed induction	10.1	Failed MT ASTS	S	1900	9.2	1	8	—

26	G2A1 with thick meconium in early labor	8.7	Failed MT ASTS	S	1800	9.4	2	8	—

36	G5A2P2L2 with 36 wks with previous 2 LSCS	9.8	Failed MT ASTS	S	1600	9.4	1	10	Fever for 3 days

37.5	G7AOP6L3 with 38 wks with placenta previa with APH	7.6	A failed MT STS	S	1700	9.3	4 with 10 units of FFP	8	

28.5	G3A1P1L1 with 39 wks with prolonged LPV with failed induction	12.2	Failed MT ASTS	S	1400	11.3		16	

30	G4A1P2L1 with 37 wks with previous 2 LSCS	8	Failed MT ASTS	S	1300	9.7		10	

27	G2P1L1 with 39	9.8	Failed MT ASTS	S	1600	9.4	1	8	Superficial infection

O: outcome; S: successful; EBL: estimated blood loss; MBLS: modified B-Lynch suture; ASTS: Alternate Sequential Suture Tightening; APH: antipartum hemorrhage; MT: medical treatment; PP: placenta previa; PROM: prelabor rupture of membrane; CPD: cephalopelvic disproportion; LPV: leaking per vagina; FFP: fresh frozen plasma; LSCS: lower segment cesarean section.
